# N-acetylcysteine addition after vitrification improves oocyte mitochondrial polarization status and the quality of embryos derived from vitrified murine oocytes

**DOI:** 10.1186/s12917-018-1743-2

**Published:** 2019-01-17

**Authors:** Elvira Matilla, Francisco Eduardo Martín-Cano, Lauro González-Fernández, Francisco Miguel Sánchez-Margallo, Ignacio Santiago Álvarez, Beatriz Macías-García

**Affiliations:** 1 Jesús Usón Minimally Invasive Surgery Centre, Carretera N-521, km. 41,8, 10071 Cáceres, Spain; 2 Department of Physiology, Faculty of Nursing and Occupational Therapy, University of Extremadura, Avda. de la Universidad s/n, 10003 Cáceres, Spain; 3 Research Group of Intracellular Signaling and Technology of Reproduction (SINTREP), Institute of Biotechnology in Agriculture and Livestock (INBIO G+C) University of Extremadura, Avda. de la Universidad s/n, 10003 Cáceres, Spain; 4 Department of Cell Biology, School of Life Sciences, University of Extremadura, Avda. de Elvas, 6006 Badajoz, Spain

**Keywords:** Mouse, N-acetylcysteine, Oocyte, Vitrification

## Abstract

**Background:**

Vitrification is the safest method to cryopreserve oocytes, however the process alters mitochondrial function resulting from increased reactive oxygen species (ROS) production. Our aim was to alleviate ROS stress in vitrified mice oocytes using N-acetylcysteine (NAC at 1 mM), to improve the oocyte’s developmental competence.

**Results:**

Hence, four experimental groups were compared: fresh oocytes (F-C), vitrified oocytes (V-C), NAC addition prior to oocyte vitrification (V-NAC-Pre) and NAC addition after vitrification (V-NAC-Post). V-NAC-Pre and V-NAC-Post exhibited higher levels of mitochondrial polarization compared to vitrified oocytes (36.5 ± 3.1, 37.7 ± 1.3 and 27.2 ± 2.4 measured as the spatial coefficient of variation/oocyte respectively, mean ± SEM; *p* < 0.05). However, ROS production increased in vitrified oocytes added with NAC compared to the vitrified control (1124.7 ± 102.1 [V-NAC-Pre] and 1063.2 ± 82.1 [V-NAC-Post] vs. 794.6 ± 164.9 [V-C]; arbitrary fluorescence units/oocyte, mean ± SEM; *p* < 0.05). ATP significantly decreased in V-NAC-Pre compared to V-NAC-Post oocytes (18.5 ± 6.9 vs. 54.2 ± 4.6 fmol/oocyte respectively, mean ± SEM; *p* < 0.05), and no differences were observed between V-NAC-Post, F-C and V-C groups. Blastocyst rates derived from F-C oocytes was higher than those derived from V-NAC-Pre (90.7 ± 1.8 vs. 79.1 ± 1.8, respectively, mean % ± SEM,; *p* < 0.05) but similar to those derived from V-NAC-Post (90.7 ± 1.8, mean % ± SEM, *p* > 0.05). Total blastomere count of blastocysts derived from V-NAC-Post after in vitro fertilization (IVF) was higher than embryos produced from V-C.

**Conclusions:**

The addition of NAC after vitrification improves the quality of vitrified mature murine oocytes while its addition prior to vitrification is advised against.

## Background

Mitochondria are core organelles in the oocyte as they determine their meiotic and developmental competence [1]. These organelles are in charge of energy production in the form of ATP through the oxidative phosphorylation chain giving as a result a controlled Reactive Oxygen Species (ROS) production [2]. Specifically, mitochondrial membrane potential is an important parameter defining mitochondrial and cellular status [3]. In mature oocytes, mitochondrial size, function and overall number, are critical factors associated to successful fertilization and subsequent embryo development [4]. In fact, during the progression of the premature oocyte within the primordial follicle to the mature oocyte (metaphase II or MII), the number of mitochondria vividly increases due to the high energy demands required for oocyte fertilization and subsequent cleavage [5].

It has to be mentioned that the oocyte represents the female germline and its long term maintenance enables a flexible use for assisted reproductive techniques [6]. In this sense, vitrification is the most rapid and safest method to cryopreserve oocytes, but during this process the mitochondrial membrane potential is altered resulting in an oxidative stress due to gradual accumulation of free radicals, causing a decrease in the survival rate and developmental competence of the oocytes, fertilization and embryo development [7–9]. In addition, cryopreservation severely affects the oocyte’s mitochondrial status and function, which has been associated with implantation failure in IVF-derived embryos [4, 10]. However, such dysfunctions do not induce evident morphological abnormalities that could help to select the best embryos to transfer [11].

Therefore, exogenous antioxidant addition could help to alleviate excessive ROS production supporting mitochondrial function [4]. Supplementation of media with antioxidants could possibly help to improve gamete quality and fortify the developing embryo [12] as previously shown in vitrified-warmed bovine mature oocytes [13]. However, the appropriate antioxidants and concentrations required still remain an ongoing area of research [14]. Some antioxidants such as Glutathione (GSH) have been reported to mitigate the toxic effects of oxidative stress when added to the vitrification medium at different time points during murine oocyte vitrification [15]. For example, in mice, it has been demonstrated that supplementation with a glutathione donor prior to vitrification improves the cryotolerance of MII oocytes [16].

In this sense, the use of N-acetylcysteine or NAC (a precursor of GSH) during oocyte cryopreservation could be advantageous as it actively scavenges free radicals increasing oocyte quality in aged mice and in vitrified immature murine oocytes [17, 18]. Thus, the aim of the present study was to investigate the impact of NAC supplementation to mature murine oocytes before and after vitrification as well as its effect on ATP production, mitochondrial polarization, ROS production and embryo development and quality after IVF.

## Results

### Mitochondrial polarization analysis

**Fig. 1:**
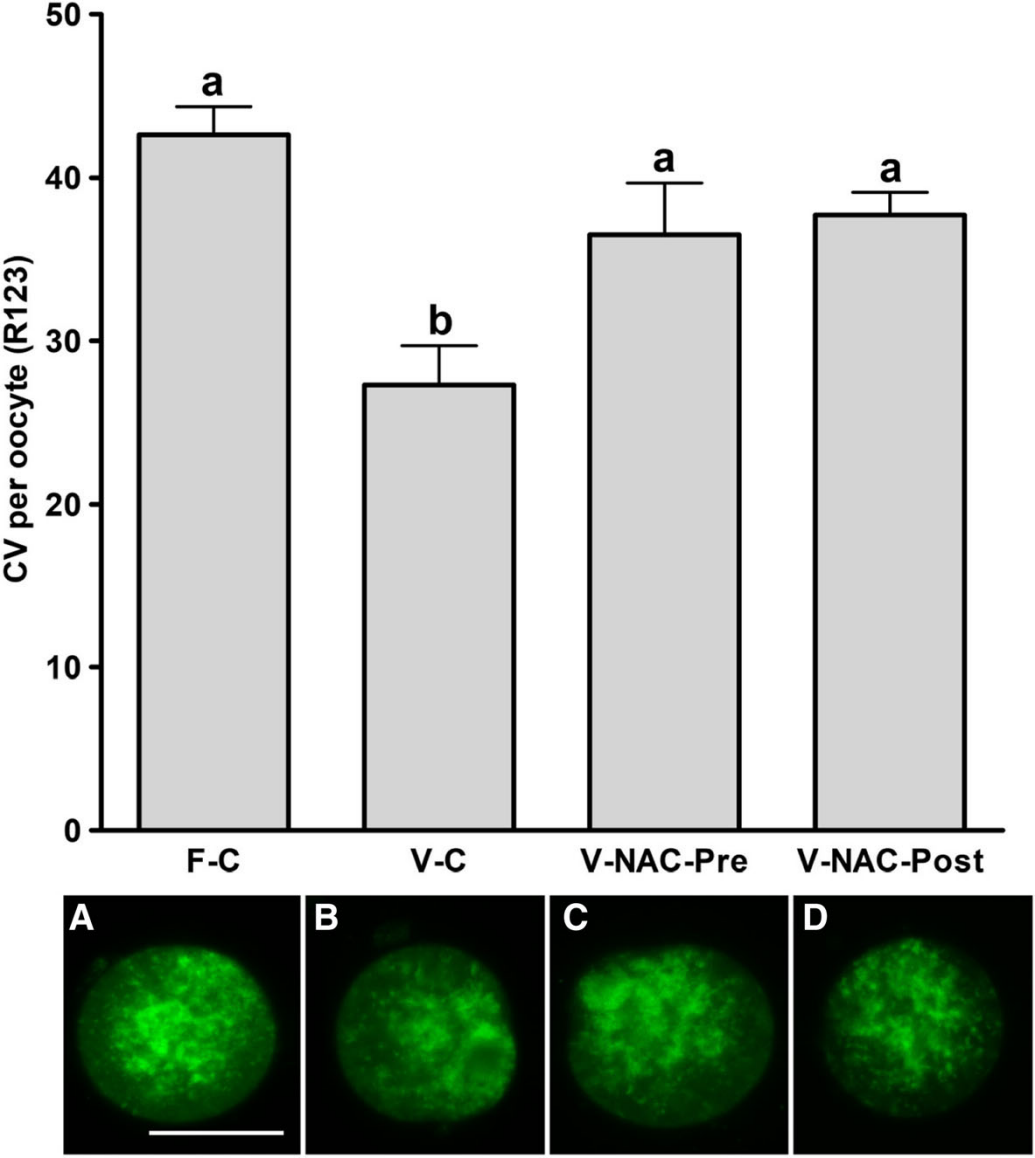
Mitochondrial polarization status of fresh and vitrified murine oocytes in presence or absence of NAC. The groups studied were: F-C: oocytes were cultured in KSOM medium for 2 h prior to IVF; V-C: oocytes were cultured in KSOM medium for 2 h prior to vitrification. After warming, oocytes were allowed to recover in KSOM medium for 2 h and subjected to IVF; V-NAC-Pre: oocytes were cultured in KSOM medium supplemented with 1 mM NAC for 2 h prior to vitrification. Warmed oocytes were allowed to recover for 2 h in KSOM medium before IVF; V-NAC-Post: oocytes were cultured in KSOM medium for two hours prior to vitrification. Warmed oocytes were allowed to recover for 2 h in KSOM medium added with 1 mM NAC before IVF. Representative micrographs of each treatment are provided below each bar and the scale represents 40 μm. Bars bearing different letters differ statistically (*p* < 0.05); values are expressed as the mean ± SEM

When all the treatments were compared between them, the vitrified control group exhibited the lowest mitochondrial polarization compared to the rest of the treatments (Fig. 1; *p* < 0.05). Interestingly, NAC addition prior to and after vitrification resulted in similar oocyte mitochondrial polarization compared to the fresh control group (36.5 ± 3.1, 37.7 ± 1.3 and 42.6 ± 1.7, coefficient of variance or CV/oocyte respectively; *p* > 0.05) (Fig. 1).

### ROS production assessment

**Fig. 2:**
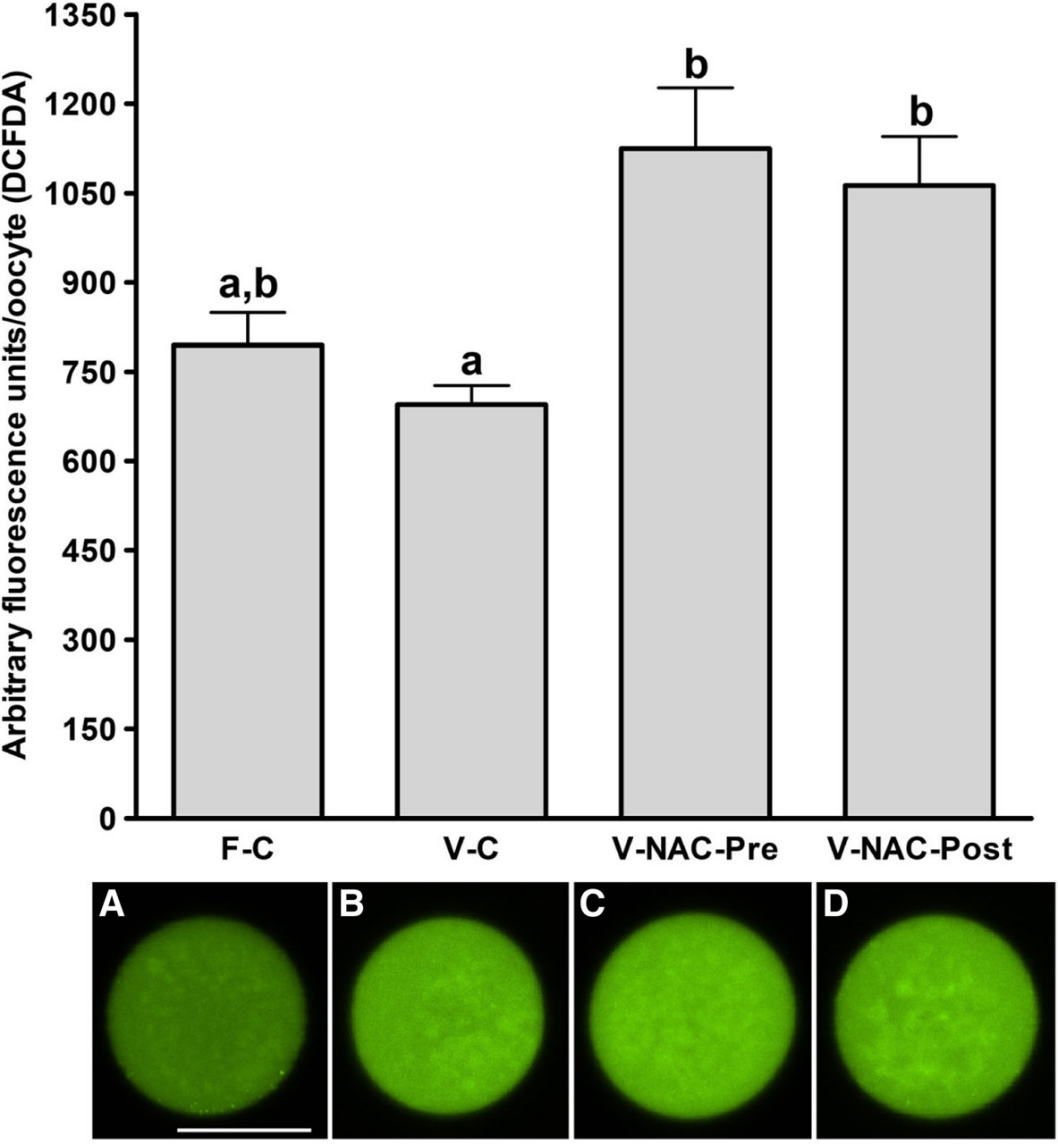
Assessment of ROS production in fresh and vitrified murine oocytes in presence or absence of NAC. The groups studied were: F-C: oocytes were cultured in KSOM medium for 2 h prior to IVF; V-C: oocytes were cultured in KSOM medium for 2 h prior to vitrification. After warming, oocytes were allowed to recover in KSOM medium for 2 h and subjected to IVF afterwards; V-NAC-Pre: oocytes were cultured in KSOM medium supplemented with 1 mM NAC for 2 h prior to vitrification. Warmed oocytes were allowed to recover for 2 h in KSOM before IVF; V-NAC-Post: oocytes were cultured in KSOM medium for 2 h prior to vitrification. Warmed oocytes were allowed to recover for 2 h in KSOM medium added with 1 mM NAC before IVF; representative micrographs of each treatment are provided below each bar and the scale represents 40 μm. Bars with different letters differ statistically (*p* < 0.05); values are expressed as mean ± SEM

After vitrification overall ROS production increased when NAC was added prior to or after vitrification compared to the vitrified control group (*p* < 0.05); interestingly, although ROS production was higher when NAC was added, this increase was not statistically significant compared to fresh control group (695.3 ± 32.1 [control] vs. 1124.7 ± 102.1 [prior to vitrification]; 1063.2 ± 82.1 [after vitrification], arbitrary fluorescence units/oocyte, mean ± SEM; *p* > 0.05) (Fig. 2).

### Determination of ATP content

**Fig. 3:**
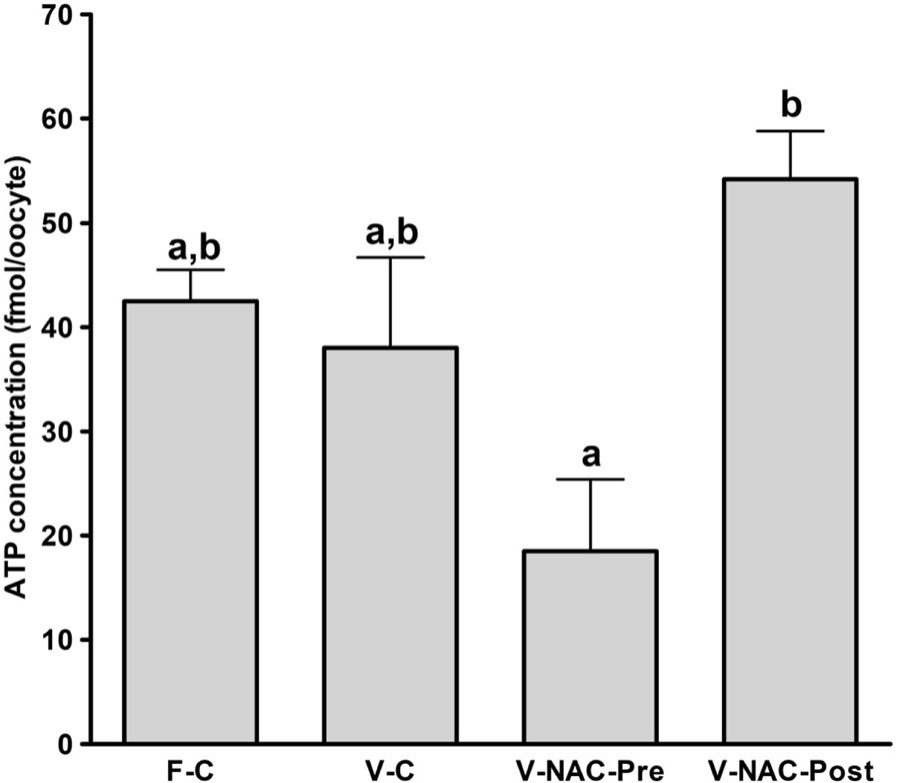
ATP content of fresh and vitrified oocytes in presence or absence of NAC. The groups studied were: F-C: oocytes were cultured in KSOM medium for 2 h prior to IVF; V-C: oocytes were cultured in KSOM medium for 2 h prior to vitrification. After warming, oocytes were allowed to recover in KSOM medium for 2 h and subjected to IVF; V-NAC-Pre: oocytes were cultured in KSOM medium supplemented with 1 mM NAC for two hours prior to vitrification. Warmed oocytes were allowed to recover for 2 h in KSOM medium before IVF; V-NAC-Post: oocytes were cultured in KSOM medium for 2 h prior to vitrification. Warmed oocytes were allowed to recover for 2 h in KSOM medium added with 1 mM NAC before IVF. Bars with different letters differ statistically (*p* < 0.05); values are expressed as mean ± SEM

Our results showed that the fresh and vitrified controls groups did not differ in their ATP content (42.5 ± 3.0 vs. 38 ± 8.7 fmol/oocyte respectively, mean ± SEM; *p* > 0.05). However, addition of NAC significantly decreased ATP content when NAC was added prior to vitrification compared to NAC addition after vitrification (18.5 ± 6.9 vs. 54.2 ± 4.6 fmol/oocyte respectively, mean ± SEM; *p* < 0.05) (Fig. 3).

### Development to the blastocyst stage and total blastomere count

**Fig. 4:**
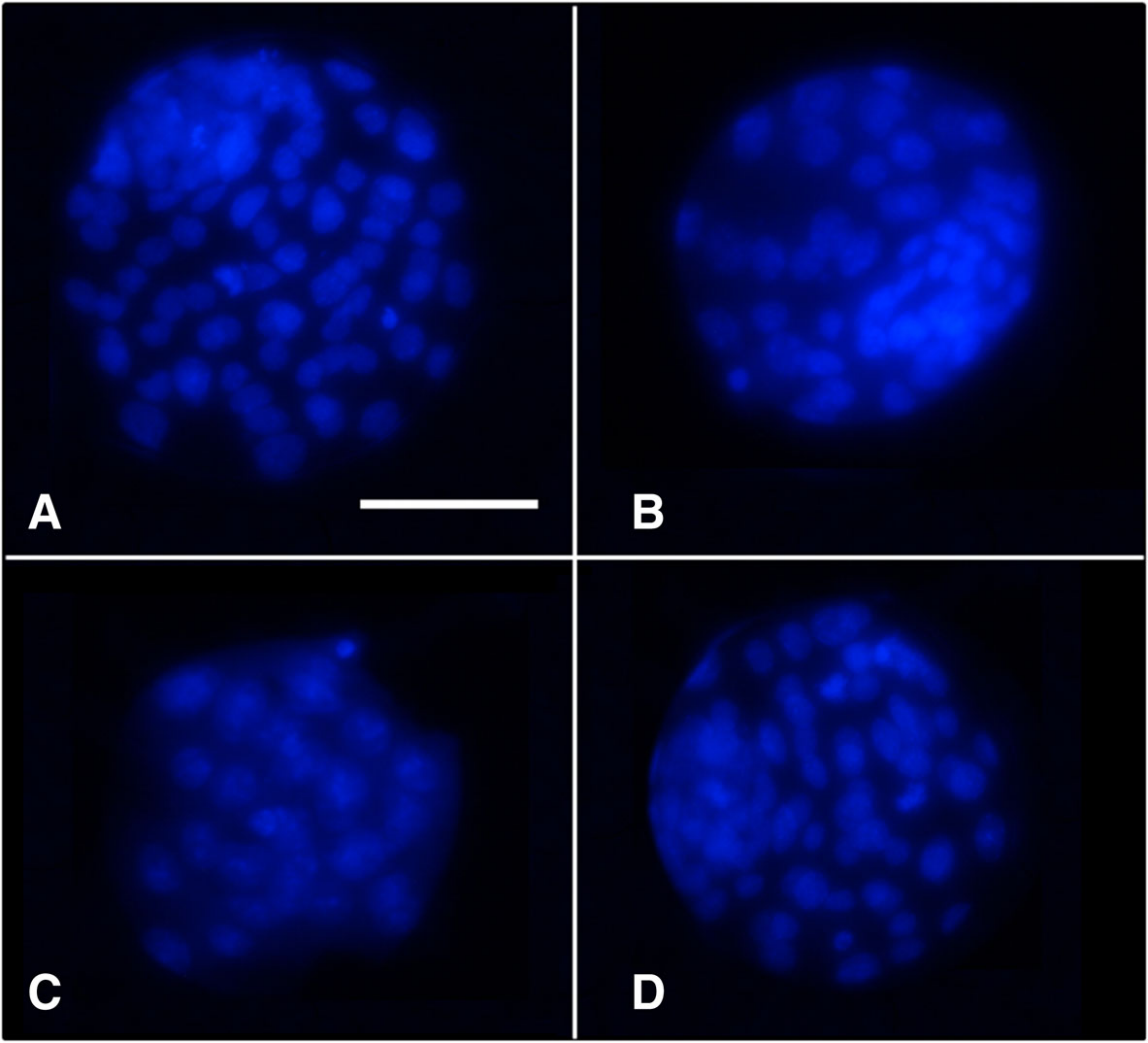
Representative micrographs of expanded blastocysts stained with Hoechst 33342 derived from fresh and vitrified murine oocytes after IVF. The letters in each panel represent: **a**) Fresh oocytes cultured in KSOM medium for 2 h; **b**) Vitrified oocytes cultured in KSOM medium for 2 h after warming; **c**) oocytes cultured in KSOM medium supplemented with 1 mM of NAC for 2 h prior to vitrification and cultured in KSOM medium for 2 h after warming**; d**) Vitrified oocytes cultured in KSOM medium supplemented with 1 mM of NAC for 2 h after warming. The scale represents 100 μm; the micrographs were taken using a 40 × objective

Our results showed statistically significant differences between blastocyst rates of embryos derived from fresh oocytes and those obtained from vitrified oocytes added with 1 mM NAC prior to vitrification (90.7 ± 1.8 vs. 79.1 ± 1.8, respectively, mean % ± SEM; *p* < 0.05) (Table 1). Also, statistically significant differences were found in the blastocyst rates between vitrified oocytes added with NAC prior to vitrification and oocytes added with NAC after vitrification (79.1 ± 1.8 vs. 90.1 ± 1.8, respectively, mean % ± SEM; *p* < 0.05) (Table 1). Interestingly, the total cell number of blastocyst derived from the vitrified NAC-Post group significantly increased compared to the vitrified control group (76.8 ± 4.1 vs. 58.9 ± 2.5 total cell number respectively, mean ± SEM; *p* < 0.05)(Table 1 and Fig. 4).

**Table 1:** Development to the blastocyst stage and total blastomere number

Oocyte treatment	n	Blastocyst rate (%)	n	Cell count
Fresh control	67	90.7 ± 1.8^a^	12	86.8 ± 2.5^a^
Vitrified Control	92	85.9 ± 1.5^a,b^	15	58.9 ± 2.5^b^
Vitrified NAC-Pre	81	79.1 ± 1.8^b^	12	36.7 ± 2.3^c^
Vitrified NAC-Post	80	90.1 ± 1.8^a,c^	12	76.8 ± 4.1^d^

Development to the blastocyst stage and total cell number of embryos obtained by IVF from fresh or vitrified murine oocytes in presence or absence of NAC. The groups studied were: F-C: oocytes were cultured in KSOM medium for 2 h prior to IVF; V-C: oocytes were cultured in KSOM medium for 2 h prior to vitrification. After warming, oocytes were allowed to recover in KSOM medium for 2 h and subjected to IVF afterwards; V-NAC-Pre: oocytes were cultured in KSOM medium supplemented with 1 mM NAC for 2 h prior to vitrification. Warmed oocytes were allowed to recover for 2 h in KSOM medium before IVF; V-NAC-Post: oocytes were cultured in KSOM medium for 2 h prior to vitrification. Warmed oocytes were allowed to recover for 2 h in KSOM medium added with 1 mM NAC before IVF. Values bearing different letters in the same column differ statistically (*p* < 0.05); values are expressed as the mean ± SEM.

## Discussion

Vitrification and warming significantly affects the developmental competence of the oocytes [2, 19]. Among other deleterious effects, vitrification induces mitochondrial depolarization of the oocyte [8] decreasing its ATP content and enhancing ROS production [20]. In addition, it is known that the mitochondrial network is highly vulnerable to temperature fluctuations and vitrification is known to alter mitochondrial distribution of mature oocytes [21]. Regarding mitochondrial distribution, a previous report has determined that a fine homogeneous pattern of active mitochondria could be an indicator of poor developmental competence in ovine and murine oocytes [22, 23]. In our experiments a higher mitochondrial polarization level was observed in fresh oocytes compared to their vitrified counterparts, and NAC addition prior to or after vitrification enhanced mitochondrial polarization to the level observed in fresh oocytes (Fig. 1). Our results are in agreement with those of Lei et al. (2014) who found that vitrification induced an abnormal mitochondrial distribution and decreased mitochondrial activity in mouse oocytes [21]. In addition, NAC addition prior to and after vitrification significantly increased mitochondrial polarization coinciding with the results reported by Yue et al. (2016) for immature mouse oocytes [18]. These results suggest that the assessment of mitochondrial polarization status using the CV can be used to objectively evaluate mitochondrial distribution. However, in contrast with the observations by Nagai et al. [23], we did not observe any correlation among mitochondrial distribution and the oocyte’s developmental competence (Table 1 and Fig. 1). Hence, further experiments were carried out to determine ROS and ATP production for each experimental group to further assess oxidative stress and mitochondrial function.

It is known that vitrification alters the endogenous antioxidant systems which are responsible for the increase in the intracellular ROS levels [7]. In porcine oocytes, it has been demonstrated that ROS production increases after oocyte vitrification [24]. To alleviate this oxidative burst, antioxidant addition to the medium prior to vitrification [24] or during the recovery culture after warming [13] has been considered. Interestingly, our results showed that ROS production in vitrified-warmed oocytes was similar to that of fresh oocytes, while oocyte vitrification in presence of NAC enhanced ROS production. The homogeneity in ROS production of fresh and vitrified oocytes is in contrast with those of Tatone et al. (2011) who showed that vitrification enhanced ROS production in murine oocytes. This divergence can be explained because of the different mice strains used (CD-1 in their work vs. B6D2 in ours) or could be due to the fact that, in our setting, oocytes are incubated for moderate times prior to and after vitrification, inducing a slight oocyte aging. In this sense, it has been demonstrated that in vitro oocyte ageing induces lower ROS production after vitrification (that is similar to aged fresh oocytes) compared to young oocytes [25]. Even when this could seem to be a matter of concern, the incubation times used in our experimental design (maximum of 5 h when NAC was added after vitrification) have been shown not to detrimentally affect the oocyte’s developmental competence [26].

Regarding the ROS increase observed in the vitrified NAC-added groups compared to the vitrified control group (Fig. 2), this result could be explained by the biphasic effect of NAC previously described in human primary gingival fibroblasts (hPGF). The report by Spagnuolo et al. (2006) described that the addition of 1 mM of NAC in presence of the pro-oxidant compound 2-hydroxyethyl methacrylate to hPGF effectively prevents ROS formation for the 5 and 10 mM dosages along the time (up to 6 h) [27]. Surprisingly, when 1 mM NAC was used, this antioxidant was capable of reducing overall ROS production after 2 h but exhibited a pro-oxidant activity at 6 h [27]. Moreover, it has been demonstrated that reduced intracellular ROS levels do not necessarily correlate with improved embryo cryotolerance or enhanced developmental competence in bovine, not being ROS production a reliable indicator of cryopreservation-induced damage or in vitro fertilization outcomes [28].

In view of these results, ATP production was measured as it is known that mitochondrial damage affects the oocyte’s developmental competence impairing ATP production and increasing embryo arrest [2]. Interestingly, ATP production remained unchanged in vitrified oocytes compared with fresh oocytes, contrasting with previous research that demonstrated an ATP decline after oocyte cryopreservation [29, 30]. However, ATP production significantly decreased when NAC was added prior to vitrification compared with NAC added after vitrification, suggesting that its addition before vitrification is not indicated. Furthermore, the fact that the blastocyst rate in this group after IVF was the lowest, suggests that NAC addition prior to vitrification exerts a toxic effect on mitochondrial function. It has to be noted that in our experiments the number of blastomeres significantly decreased after vitrification compared to the fresh counterparts, as also demonstrated for mice cryopreserved embryos [31]. Notably, when NAC was added after vitrification, the number of blastomeres significantly increased compared to the vitrified control group or vitrified oocytes added with NAC prior to cryopreservation, demonstrating an improvement on embryo quality [32, 33]. Although total blastomere number was lower in vitrified embryos added with NAC after vitrification than in the fresh control group, total cell number was almost doubled compared to vitrified embryos added with NAC before vitrification (Table 1 and Fig. 4). These results together with the lower developmental competence and diminished ATP production of the oocytes added with NAC before vitrification demonstrate that NAC should be added to murine oocytes after vitrification. In addition, ROS production or mitochondrial polarization are not reliable indicators of mitochondrial status, being ATP content determination a more adequate parameter. Although previous reports showed that glutathione donors improve murine oocyte’s cryotolerance and maintain ATP content [16], in this work ATP was measured right after oocyte warming while in our work oocytes were allowed to re-equilibrate, possibly explaining the observed differences.

## Conclusions

In conclusion, supplementation of mice oocytes with NAC after vitrification improves mitochondrial status and total cell number in expanded blastocysts. NAC addition prior to cryopreservation is advised against, as it impairs the ATP content of murine oocytes and erodes their developmental competence. More research is necessary to understand the mechanism by which NAC exerts its effects on murine oocytes, the optimum dosages/incubation times and to develop and improve post-warming equilibration media by NAC supplementation. Our findings could be of clinical interest and more studies are required to test if our results can be extrapolated to other domestic species.

## Methods

### Reagents

All the reagents were purchased from Sigma-Aldrich (Barcelona, Spain) unless otherwise stated.

### Animals

**Fig. 5:**
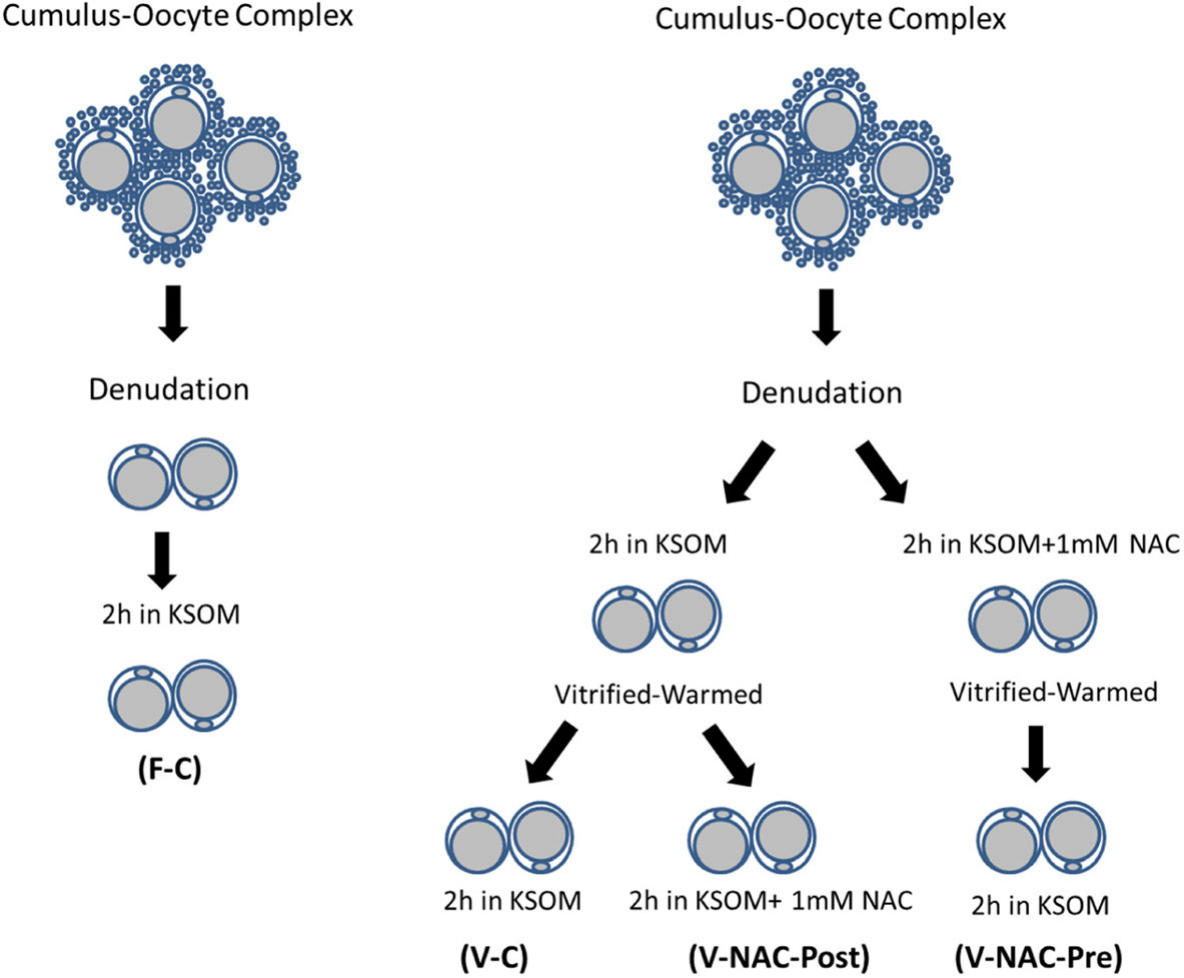
Diagram explaining the experimental design used in our study. F-C: MII denuded oocytes were cultured in KSOM medium for 2 h prior to IVF; V-C: MII denuded oocytes were cultured in KSOM medium for 2 h prior to vitrification. After warming, oocytes were allowed to recover in KSOM medium for 2 h and subjected to IVF; V-NAC-Pre: MII denuded oocytes were cultured in KSOM medium supplemented with 1 mM NAC for two hours prior to vitrification. Warmed oocytes were allowed to recover for 2 h in KSOM medium before IVF; V-NAC-Post: MII denuded oocytes were cultured in KSOM medium for 2 h prior to vitrification. Warmed oocytes were allowed to recover for 2 h in KSOM medium added with 1 mM NAC before IVF

B6D2F1/J mice were bred at the authorized animal housing of the CCMIJU. The C57BL/6JOlaHsd females and DBA/2OlaHsd males used as parental lines were purchased from Envigo (www.envigo.com). The animals were housed under 12 h light/12 h dark cycles at a controlled temperature (19–23 °C), with free access to food and water. Females (8–12 weeks of age) were intraperitoneally (IP) injected with 8 IU of equine chorionic gonadotrophin (Veterin Corion, Divasa Farmavic) followed 49 h later by 8 IU of IP human chorionic gonadotrophin (Foligon, MSD) to trigger ovulation. Female mice were euthanized 12 h after hCG administration by cervical dislocation after which respiratory arrest and the absence of heartbeat were confirmed by palpation. Then, cumulus-oocyte complexes (COCs) were recovered from oviducts and placed in 500 μL of Human Tubal fluid (HTF) covered with mineral oil. One hour later, COCs were denuded using hyaluronidase (80 IU/mL) in M2 medium for no more than one minute and washed in M2 medium. After denudation, MII oocytes were separated in different experimental groups (Fig. 5). Once the experiments were over, the carcasses were incinerated, following the approved protocol.

### Vitrification and warming

MII denuded oocytes were equilibrated in M2 medium added with 7.5% of DMSO (v:v), 7.5% ethylene glycol (v:v) and 20% (v:v) fetal bovine serum (FBS) for 3 min. Afterwards, the oocytes were moved to a vitrification solution consisting of M2 medium supplemented with 20% FBS added with 15% ethylene glycol (v:v), 15% DMSO (v:v) and 0.5 M sucrose for 1 min. Groups of 10 to 15 oocytes were loaded in 0.25 mL French straws (IMV, L’Aigle, France) at room temperature and sealed by ultrasounds (Superultrasonic Co; Ltd. Taiwan). After that, the straws were plunged into liquid nitrogen and stored for at least 7 days. Oocytes were warmed at 37 °C for 3–4 min in M2 medium added with 0.5 M sucrose and 20% FBS and washed in M2 medium for further 3 min.

### In vitro fertilization

Male B6D2F1/J mice aged 8–12 weeks were euthanized by cervical dislocation and ventrally dissected to remove cauda epididymis. Once located, the epididymis and attached vas deferens were sectioned and transferred to a Petri dish containing 500 μL of pre-equilibrated HTF covered with mineral oil. Spermatozoa were obtained by gently pressing cauda epididymis through the vas deferens and were allowed to capacitate for 45 min at 37 °C in a 5% CO_2_/ 95% air atmosphere at 100% humidity. At the end of the incubation, sperm concentration was measured using a Makler® counting chamber (Irvine Scientific, CA, USA). Oocytes were inseminated with 2 × 10^6^ sperm/mL; gametes were co-incubated for 3 h, moved to clean KSOM medium and followed in culture until they reached the expanded blastocyst stage.

### Experimental design

Oocytes were separately allocated to different experimental groups as represented in Fig. 5. The NAC dosage used and incubation time after warming were chosen based on previous reports [34–36].

### Mitochondrial polarization status

Denuded oocytes were photosensitized using the mitochondrial-specific fluorophore Rhodamine 123 (R123) at 5 μM at 37 °C added to 4-(2-hydroxyethyl)-1-piperazineethanesulphonic acid (HEPES)-base medium containing (mM): 140 NaCl, 5 KCl, 1.5 CaCl_2_, 1 MgCl_2_, 20 glucose, 10 HEPES, with the pH adjusted (NaOH) to 7.4 for 10 min as described previously [37]. After loading, the chamber containing the oocytes was perfused for 2 min with Na^+^ -HEPES-based medium in order to remove excess of R123. Oocytes were then imaged with a confocal scanning laser microscopy (Eclipse Ti, Nikon) at 40 × magnification using a 488 nm argon laser and the emission was collected through a 525/550 nm filter following the method described by Toescu et al. (2000). This method is based on the determination of the heterogeneity of the signal from R123 as an indicator of the depolarization of the mitochondrial network. The heterogeneity is calculated by the spatial coefficient of variation (CV; standard deviation or SD/average) of the whole body fluorescence, thus, the higher the CV, the more hyperpolarized the mitochondrial network is. In our setting, in the oocytes with polarized mitochondria, the image taken contained a number of pixels with very high intensity, corresponding with mitochondria accumulating lipophilic dyes and many pixels with low intensity which coincide with the cytosolic and nuclear regions; as a result, both the SD and CV were large. In oocytes with depolarized mitochondria, the distribution of individual pixel intensities will be more homogeneous, as the mitochondrial signal will decrease and, at the same time, the signal from cytosol will increase, being their CV lower [37]. Images were acquired using NIS Elements software (Nikon, Japan) and analysed with Fiji (NIH [38]); for each oocyte the confocal section corresponding to the oocyte’s maximum diameter was considered and overall fluorescence was analysed. In these experiments 12 oocytes/treatment collected in 3 different days from 9 different animals (3 mice per session) were analyzed.

### ROS assessment

To measure ROS levels, the oocytes were transferred to HEPES base medium added with 4 μM 2′, 7′-dichlorodihydrofluorescein diacetate (DCFDA; Merck; Darmstadt, Germany) at 37 °C for 15 min in the dark. Then, the solution was removed and the oocytes (12 oocytes/treatment collected in 3 different days from 9 different animals; 3 mice per session) were washed for 1 min with HEPES-base medium. The cells were then imaged with a confocal microscope (confocal scanning laser (Nikon A1) coupled to an inverted microscope (Eclipse Ti, Nikon)) using a 480 nm argon laser; the emission was collected through a 525/550 nm filter and the intensity of the fluorescence was determined with Fiji (NIH [38]).

### Oocyte ATP content determination

ATP concentrations were determined using the ATP Bioluminescent Somatic Cell Assay Kit with minor modifications as previously described [39]. Ten to fifteen oocytes from each group were snap-frozen in a sterile microtube containing 100 μL of ultrapure water and stored at − 80 °C. The oocytes (38 to 45 per treatment) were collected at 3 different days from 12 different animals and the analysis was performed in 4 different sessions. The day of the experiment, a volume of 50 μL of ATP assay mix Working Solution was added to individual wells in an opaque 96-well plate and kept at room temperature for 3 min to allow for endogenous ATP hydrolysis. Fifty microliters of the thawed samples were mixed with Somatic Cell ATP Releasing Reagent (1:100). This mixture was transferred to the wells and the amount of bioluminescence emitted was measured immediately using a Synergy 2 plate reader (Varioskan, ThermoFisher Scientific). Background luminescence was subtracted from all readings. ATP concentration was calculated by comparison against a standard curve.

### Development to the blastocyst stage and blastocyst cell number determination

The presumptive zygotes were followed for 4 days to assess their development to the expanded blastocyst stage; the blastocyst formation rate was recorded using a stereomicroscope for each group individually. Blastocyst of each group were fixed in 4% parafomaldehyde and stained with Hoechst 33258 (2.5 μg/mL in PBS) added with 0.2% of polyvinyl alcohol (PVA) for 10 min at 37 °C in the dark. At the end of the incubation, the expanded blastocysts were mounted on glass slides using glycerol, covered with coverslips, sealed with nail polish and analyzed using a fluorescence microscope (Nikon Eclipse TE2000-S) equipped with an ultraviolet lamp. Pictures were taken and the number of blastomeres of each blastocyst was determined. Cell number was analyzed using the Fiji Image-J software (1.45q, Wayne Rasband, National Institutes of Health, USA).

### Statistical analysis

Data were tested for normality using a Shapiro–Wilk test; results are reported as mean ± standard error of the mean (SEM). Groups were compared using ANOVA on ranks due to their non-Gaussian distribution. When statistically significant differences were found, a Holm-Sidak or Dunn’s post-hoc test was used to compare pairs of values. All statistical analyses were performed using Sigma Plot software version 12.3 for Windows (Systat Software, Chicago, IL, USA). Differences among values were considered statistically significant when *p* < 0.05.

## Abbreviations

ANOVA: analysis of variance
ATP: Adenosine triphosphate
B6D2 or B6D2F1/J: Cross between C57BL/6 J (B6) female x DBA/2 J (D2) male
CD-1: Crl: CD1(ICR) mouse strain
CV: Standard deviation/average
F-C: Fresh control oocytes
GSH: Glutathione
HTF: Human tubular fuid
IVF: In vitro fertilization
KSOM: Potassium-supplemented simplex optimized medium
MII: Metaphase II
NAC: N-acetylcysteine
ROS: Reactive oxygen species
SD: standard deviation
SEM: Standard error of the mean
V-C: Vitrified control oocytes
V-NAC-Post: NAC addition after oocyte vitrification
V-NAC-Pre: NAC addition prior to oocyte vitrification
